# Mayaro Virus Infects Human Brain Cells and Induces a Potent Antiviral Response in Human Astrocytes

**DOI:** 10.3390/v13030465

**Published:** 2021-03-11

**Authors:** Michèle Bengue, Pauline Ferraris, Jonathan Barthelemy, Cheikh Tidiane Diagne, Rodolphe Hamel, Florian Liégeois, Antoine Nougairède, Xavier de Lamballerie, Yannick Simonin, Julien Pompon, Sara Salinas, Dorothée Missé

**Affiliations:** 1 MIVEGEC, Université de Montpellier, IRD, CNRS, 34394 Montpellier, France; michele.bengue@ird.fr (M.B.); pauline.ferraris@gmail.com (P.F.); c.diagne@icloud.com (C.T.D.); rodolphe.hamel@ird.fr (R.H.); florian.liegeois@ird.fr (F.L.); julien.pompon@ird.fr (J.P.); 2 Pathogenesis and Control of Chronic Infections, Inserm, Université de Montpellier, Etablissement Français du Sang, 34394 Montpellier, France; jonathan.barthelemy@inserm.fr (J.B.); yannick.simonin@umontpellier.fr (Y.S.); 3 Unité Des Virus Emergents (UVE, Aix Marseille Université, IRD 190, Inserm 1207, IHU Méditerranée Infection), 13005 Marseille, France; antoine.nougairede@univ-amu.fr (A.N.); xavier.de-lamballerie@univ-amu.fr (X.d.L.)

**Keywords:** mayaro, chikungunya, alphavirus, arbovirus, neural progenitors, pericytes, astrocytes, neurotropism

## Abstract

Mayaro virus (MAYV) and chikungunya virus (CHIKV) are known for their arthrotropism, but accumulating evidence shows that CHIKV infections are occasionally associated with serious neurological complications. However, little is known about the capacity of MAYV to invade the central nervous system (CNS). We show that human neural progenitors (hNPCs), pericytes and astrocytes are susceptible to MAYV infection, resulting in the production of infectious viral particles. In primary astrocytes, MAYV, and to a lesser extent CHIKV, elicited a strong antiviral response, as demonstrated by an increased expression of several interferon-stimulated genes, including *ISG15*, *MX1* and *OAS2*. Infection with either virus led to an enhanced expression of inflammatory chemokines, such as CCL5, CXCL10 and CXCL11, whereas MAYV induced higher levels of IL-6, IL-12 and IL-15 in these cells. Moreover, MAYV was more susceptible than CHIKV to the antiviral effects of both type I and type II interferons. Taken together, this study shows that although MAYV and CHIKV are phylogenetically related, they induce different types of antiviral responses in astrocytes. This work is the first to evaluate the potential neurotropism of MAYV and shows that brain cells and particularly astrocytes and hNPCs are permissive to MAYV, which, consequently, could lead to MAYV-induced neuropathology.

## 1. Introduction

Arthropod-borne viruses have been identified in all continents as emerging and re-emerging etiologic agents, causing illness in humans and domestic animals, thereby becoming a serious challenge to public health [1]. The emergence of these viruses and their epidemic potential is thought to be favored by environmental factors, international travels, urban development, in addition to climate and ecological changes along with the spread of vectors and reservoirs [2]. Mayaro virus (MAYV) is an alphavirus principally maintained in a cycle involving *Haemagogus janthinomys* as a main vector and non-human primates as primary hosts [3]. Amongst several others potential vectors, the major anthropophilic urban mosquitoes (*Aedes aegypti* and *Aedes albopictus*) and some *Anopheles* species have been shown to be able to transmit MAYV [4,5,6]. It was first identified in 1954 from rural workers in Trinidad and Tobago and thought to be limited to the South American continent causing sporadic outbreaks next to the forest environment [7,8]. However, the outbreak reported in Haiti in 2015 suggests that the virus is extending its reach, along with human to human transmission [9,10]. More importantly, an epidemiological alert was announced by the Pan American Health Organization in 2019 due to the rise in outbreaks and the plasticity of the virus [11].

In addition, imported cases have been described in various part of the world includingin Europe and the USA [7]. MAYV presents nonspecific symptoms resembling “dengue-like” syndrome, with as common clinical manifestations fever, polyarthralgia/polyarthritis, rash and occasional myalgia, vomiting and diarrhea. The symptoms are generally self-limited [7,12]. Phylogenetic studies have been permitted to classify MAYV in three genotypes (D, L and N) depending on their geographic distribution [7]. 

MAYV is a single stranded RNA-positive sense virus belonging to the Semliki Forest Complex of the alphavirus genus and the Togaviridae family [7,12]. Viruses of this genus are divided into two groups: those causing encephalopathy, such as Venezuelan, Western and Eastern Equine Encephalitis viruses (endemic to Americas), or arthritis, including MAYV, CHIKV, Sindbis virus (SINV) and Ross River virus (dispersed all over the world) [13]. 

Some Old World alphaviruses, such as MAYV, are known for their capacity to cause chronic polyarthralgia and/or polyarthritis [14]. MAYV is phylogenetically related to chikungunya virus (CHIKV) despite their different continental origins [12]. However, it has been reported that CHIKV has both arthritogenic and neurotropic properties, with neurological impairment being one of the most severe complications following infection [15,16]. The first reports of CHIKV affecting the central nervous system (CNS) emerged during epidemics in Thailand and India [17,18]. Neuro-chikungunya is mainly associated with symptoms such as meningo-encephalopathy, seizures, encephalomyelitis, Guillain–Barre syndrome and optic neuritis [19,20]. CHIKV-associated neurological affections have been described in neonates, children and elderly people with long-term sequelae sometimes resulting in death [21,22,23,24]. Based on in vivo and in vitro studies, two hypotheses on the pathophysiological mechanisms underlying neurological complications of CHIKV infection have been proposed. Firstly, the virus could directly infect the CNS through entry via the choroid plexus, resulting in blood–brain barrier (BBB) damage [24]. Secondly, the neurological disorders could result from an uncontrolled inflammatory response, which is known to be harmful for CNS cells [25,26]. In contrast to CHIKV, rare cases of CNS disorders due to MAYV infection have been described [27,28], but information on the molecular mechanisms that are involved in MAYV access to the CNS in humans is lacking. 

Pericytes and astrocytes are important players of the CNS in the regulation of neuronal and neurovascular homeostasis and inflammation [29,30]. Pericytes are key BBB components but are also involved in neuroinflammatory responses [29], while astrocytes are involved in immune surveillance, and their ability to produce neurotrophic factors and anti-inflammatory cytokines and to eliminate toxins highlights their prominent role in the defense of the brain against the invasion of pathogens [31,32]. However, those same pathogens, including arboviruses, can potentially target both astrocytes [32,33] and pericytes [33,34], thereby modulating their function.

To better characterize the neurovirulence of MAYV, and compare it to CHIKV, we evaluated the ability of both viruses to infect human brain cells, including neural progenitors (hNPCs), pericytes and astrocytes, that mediate early antiviral responses to pathogens invading the CNS [35]. We show that MAYV efficiently replicates in CNS cells and induces an antiviral response that differs from that of CHIKV in primary human astrocytes.

## 2. Materials and Methods

### 2.1. Virus, Cells and Reagents

The following low passage viral strains were used in this study: Haiti-1/2015 MAYV strain was isolated in 2015 in Haiti (Genotype L; GenBank accession number KX496990) [36] and CHIKV LR2006_OPY1 strain was isolated in 2006 in La Réunion Island from a viremic patient. These viruses were propagated in C6/36 *Ae. albopictus* cells at 28 °C in Dulbecco’s modified Eagle’s medium (DMEM; Invitrogen, Carlsbad, CA, USA) supplemented with 10% fetal bovine serum (FCS; Lonza, Basel, Switzerland) to generate viral stocks of different titers. Mock-infected supernatants were obtained from C6/36 cells that had been subjected to the same procedure in the absence of MAYV or CHIKV. Primary human pericytes and astrocytes were purchased from ScienCell^TM^ and maintained according to the manufacturer’s instructions. Cells were cultured on poly-d-lysine-coated plates and were used between passage 2 and 4. Lhumes cells (hNPCs) were cultured according to established protocols [37] in advanced DMEM:F12 medium complemented with N2 supplement, Glutamax (ThermoFischer Scientific, Waltham, MA, USA), hbFGF 160µg/mL, Peprotech, Rocky Hill, NJ, USA), Penicillin/Streptomycin and Tetracycline (ThermoFischer Scientific, Waltham, MA, USA). Cells were cultured on poly-d-Ornithine/fibronectin/laminin-coated plates. Human recombinant type I and type II interferons (IFN) were purchased from R&D Systems.

### 2.2. Infection of hNPCs, Astrocytes and Pericytes 

Cells were seeded in six-well plates and propagated until 70–80% confluence (2.5 × 10^5^ cells). The three cell types were infected with MAYV or CHIKV at the indicated multiplicity of infection (MOI), or mock-infected supernatants from C6/36 cells as a control, at 37 °C with gentle agitation. After internalization for two hours (h), the cultures were washed twice with PBS and one ml of growth medium was added. The cells were maintained at 37 °C in a 5% CO_2_ atmosphere, and at the indicated time points post-infection, aliquots of supernatants were harvested and stored at −80 °C.

### 2.3. Cell Viability Assay

Astrocytes were seeded in six-well culture plates and were infected with either MAYV or CHIKV at MOI of 5 or cultured in mock-infected C6/36 supernatant in a final volume of 1 mL of astrocyte growth medium. The ReadyProbes^®^ Cell Viability Imaging Kit (Blue/Green) (ThermoFischer Scientific, Waltham, MA, USA) was used to determine cell viability at 24 and 48h post-infection (hpi) according to the manufacturer’s recommendations. Two drops of each reagent (NucBlue^®^ Live reagent (Hoechst 33342) and NucGreen^®^ Dead reagent) were added to each well. DAPI (360/460) and GFP (504/523) filters of the EVOS^TM^ microscope (Invitrogen, Carlsbad, CA, USA) were used to visualize live cells nuclei stained in blue and dead cells stained in green, respectively. 

### 2.4. Virus Titration

A plaque assay was carried out with the harvested culture supernatants to determine the efficiency of viral replication. Vero cells (1.5 × 10^5^ cells/well) in 24-well tissue-culture plates were infected with 10-fold dilutions of virus and incubated for 2 h at 37 °C with gentle rocking. The inoculum was removed and a mix of nutriment solution with agar was added to the cultures that were subsequently incubated for 5 days. Five days later, the cells were fixed in 3.7% paraformaldehyde for 15 min, incubated with 0.1% crystal violet in 20% ethanol and stained for 1 h at room temperature (RT). Viral titers were visualized, and the number of plaque-forming units (PFU) was determined.

### 2.5. Extraction and Quantification of Viral RNA

Total viral RNA was extracted from MAYV and CHIKV-infected cells using Tri reagent (Sigma), according to the manufacturer’s protocol. The quantification of viral replication kinetics was performed at 24 and 48 hpi in hNPCs, pericytes and astrocytes. Reverse transcription of 1 µg of total RNA using Moloney murine leukemia virus (M-MLV) reverse transcriptase (Promega, Madison, WI, USA) and Maxima probe/ROX qPCR master mix (Promega, Madison, WI, USA) were used for real-time PCR, as described previously [36]. For CHIKV quantification, the following primers sets and probes were used: CHIKV (CHIV_F AAGCT(CT)CGCGTCCTTTACCAAG; CHIKV_R CCAAATTGTCC(CT)GGTCTTCCT; FAM-CCAATGTC(TC)TC(AC)GCCTGGACACCT-BHQ).

### 2.6. RT^2^ Profiler PCR Array

RT^2^ First Strand kit was purchased from Qiagen for the synthesis of the complementary DNA strand of RNA extracted from human astrocytes at 48 hpi and quantified by NanoDrop spectrophotometer following the manufacturer’s instructions. RT^2^ Profiler™ PCR Array Human Antiviral Response (PAHS-122ZG-4) was used as previously described [36]. The experiments were performed in triplicates and Ct values of target genes were normalized using Actin (ACTB), Beta-2-microglobulin (B2M) and Glyceraldehyde-3-phosphate dehydrogenase (GAPDH) housekeeping genes. 

### 2.7. Real-Time PCR Gene Expression Analysis 

Total RNA was extracted from astrocytes using Tri reagent (Sigma, St. Louis, MO, USA), and 1 µg was used to synthesize cDNA using a M-MLV reverse transcription kit following the manufacturer’s protocol (Promega, Madison, WI, USA). Real-time PCR was performed with Eva Green Master Mix (Invitrogen, Carlsbad, CA, USA). Specific primers were used to quantify the expressions of targeted genes. The applied biosystems 7300 real-time PCR system was used for the gene quantification. The cycling conditions were 95 °C for 10 min followed by 45 amplification cycles of 95 °C for 15 s, 60 °C for 20 s and 72 °C for 30 s. Primers of genes targeted are listed in Table S1. The fold change in gene expressions was quantified and normalized with glyceraldehyde-3-phosphate dehydrogenase (GAPDH) as housekeeping gene. Wilcoxon–Mann–Whitney test was used for statistical analysis.

### 2.8. Indirect Immunofluorescence Assays

For indirect immunofluorescence studies, cells were fixed with 4% PFA for 15 min at RT, permeabilized with 0.1% Triton X-100/PBS for 5 min at RT, followed by a blocking step with 2% bovine serum albumin (BSA) and 10% horse serum (blocking solution, BS) for 1h at RT. Primary and secondary antibodies were diluted in BS and incubated sequentially for 1h at RT. Mouse anti-Eastern Equine Encephalitis antibody (Pan-alpha) was purchased from Sigma-Aldrich. When indicated, cells were treated during the secondary antibody incubation with ActinGreen (ThermoFischer Scientific, Waltham, MA, USA) and Hoechst (ThermoFischer Scientific, Waltham, MA, USA) nuclei counter stain. Coverslips were mounted on glass slides with fluorescent mounting medium (Fluoroshield, Sigma) and imaged by confocal microscopy using the Zeiss SP85 confocal microscope, with 40× or 63× 1.4 NA Plan Apochromat oil-immersion objectives.

### 2.9. ELISA 

Human astrocytes were seeded in 6-well plates and infected with either MAYV or CHIKV at a MOI of 5. Mock-infected supernatants from C6/36 cells were used as a control. At 48 hpi, supernatants were collected and used for protein quantification. IFNαβ, CXCL10, IL-6, IL-12 and IL-15 protein levels were quantified using ELISA kits (Invitrogen). Human IFN-α/β R2 ELISA Kit, IP-10/CXCL10 Human Instant ELISA Kit, IL-6 Human ELISA Kit, IL-12 p40/p70 Human ELISA Kit and IL-15 Human uncoated ELISA Kit were used according to the manufacturer’s recommendations. The measurement of light absorbance was done by a microplate reader (Infinite M200PRO TECAN).

## 3. Results

### 3.1. MAYV Efficiently Infects Human Neural Progenitor Cells, Astrocytes and Pericytes

**Figure 1:**
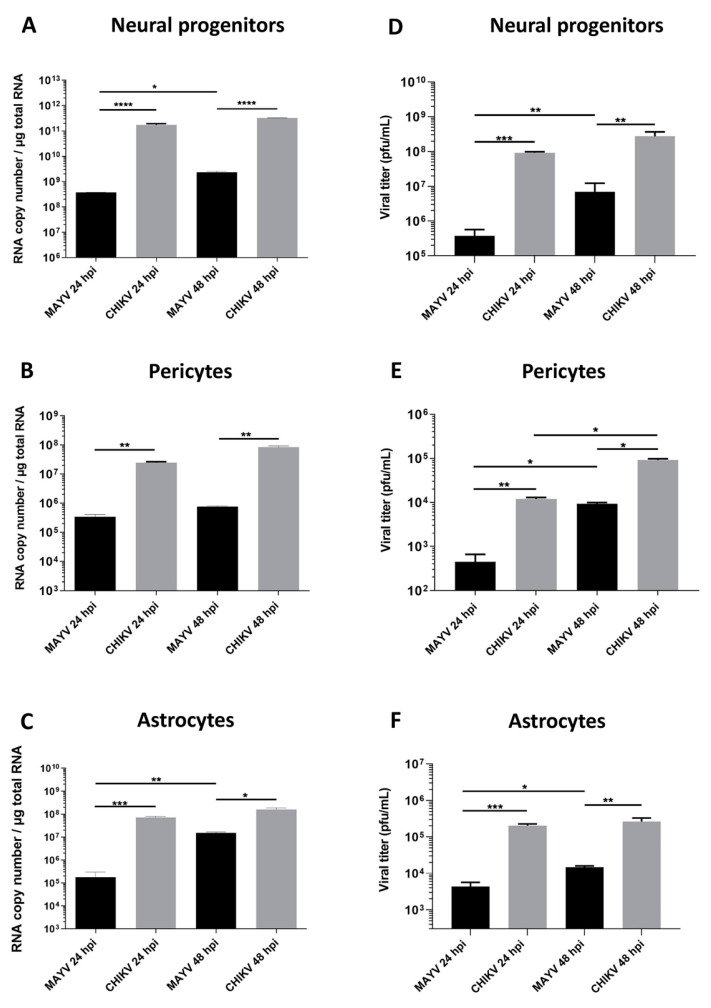
Human neural progenitors, pericytes and astrocytes are permissive to Mayaro virus (MAYV) and chikungunya virus (CHIKV) infection. Cells were infected with either MAYV or CHIKV at multiplicity of infection (MOI) 5 at 24 and 48 hpi. RT-qPCR was performed to measure viral RNA level (**A**–**C**). Infected cell supernatants were analyzed by plaque assay to quantify infectious viral particles (**D**–**F**). Statistical analyses were done from three independent experiments each performed in triplicate. Comparisons between the data at the indicated time points using a one-way ANOVA test. *p*-value; * *p* < 0.05, ** *p* < 0.01, *** *p* < 0.001, **** *p* < 0.0001.

**Figure 2:**
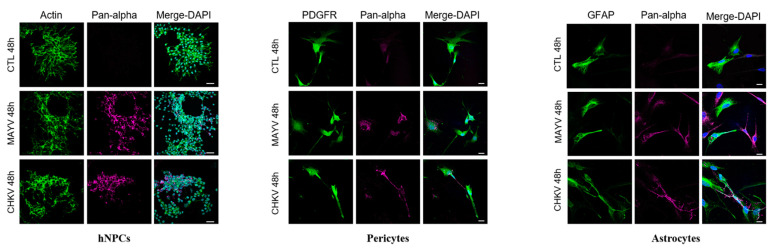
MAYV and CHIKV infect human neural progenitors (hNPCs), astrocytes and pericytes. Cells were infected with either MAYV or CHIKV at MOI 5 for 48 hpi, fixed and stained by indirect immunofluorescence for the detection of actin (hNPCs, ActinProbe, green), platelet-derived growth factor receptor (PDGFR) (pericytes, green), glial fibrillary acidic protein (GFAP) (astrocytes, green) and MAYV and CHIKV envelop protein (anti-pan-alphavirus; magenta). Nuclei are counterstained using Hoechst (blue). Scale bars equal to 30 µm (hNPCs) and 20 µm (pericytes and astrocytes). Mock-infected cells were used as control (CTL).

To evaluate and compare MAYV and CHIKV replication efficiency in human brain cells, hNPCs, primary astrocytes and pericytes were infected with either virus at MOI 5. Viral RNA and infectious viral particles were assessed at 24 and 48 hpi by RT-qPCR and plaque assay, respectively. The results show that although all cell types were infected with MAYV, the amount of viral transcripts was higher in hNPCs-infected cells compared to that observed in MAYV-infected pericytes and astrocytes (Figure 1A–C). A difference of approximately 3 logs of viral RNA copy numbers was observed between MAYV-infected hNPCs and the two other infected primary CNS cell types, as early as 24 hpi. Interestingly, there was a strong increase in the amount of MAYV transcripts in human astrocytes between 24 and 48 hpi, reaching 10^8^ RNA copies/µg of total RNA (Figure 1C). Production of infectious particles for both viruses increased in a time-dependent manner in hNPCs and pericytes, and to a lesser extent in astrocytes, although, overall, MAYV appeared to infect brain cells less efficiently than CHIKV (Figure 1). We confirmed the permissiveness of the CNS cells by performing immunofluorescence assays, using an anti-actin fluorescent probe, glial fibrillary acidic protein (GFAP, astrocyte marker) or platelet-derived growth factor receptor (PDGFR, pericyte marker), as well as pan-alphavirus antibodies. Infected hNPCs, astrocytes and pericytes showed MAYV and CHIKV labeling in all brain cells tested at 48 hpi (Figure 2). Altogether, these observations show that both viruses efficiently replicate in cells of the CNS more markedly in hNPCs.

### 3.2. MAYV Elicits a Strong Antiviral Response in Human Astrocytes

**Figure 3:**
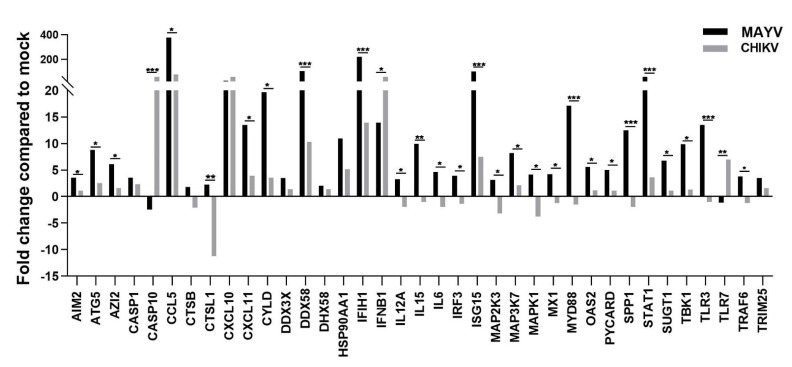
MAYV induces a strong antiviral response in human astrocytes. RNA extract from infected human astrocytes (MOI 5) at 48 hpi were subjected to RT^2^ Profiler PCR array. The modulation of antiviral response genes following MAYV or CHIKV infection is expressed as function of that with mock-infected cells. The mean values are represented by each point and two experiments (*n* = 2) was performed in triplicate. The unpaired *t*-test * *p* < 0.05, ** *p* < 0.01, *** *p* < 0.001, was employed to determine statistical significance between data obtained with MAYV or CHIKV-infected cells.

**Figure 4:**
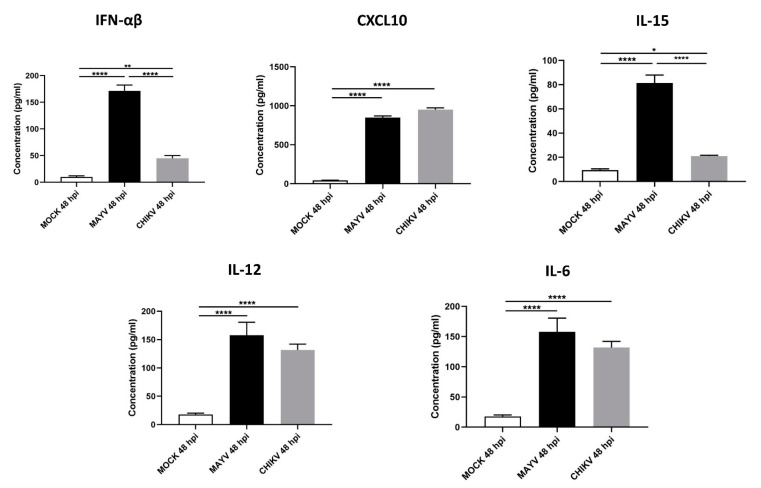
MAYV-infected human astrocytes produce high levels of inflammatory mediators. Supernatants of MAYV-, CHIKV- and mock-infected astrocytes at MOI 5 were harvested at 48 hpi and analyzed by ELISA. IFN-α/β, CXCL10, IL-15, IL12 and IL-6 cytokine levels were quantified and their values expressed in pg/mL. Statistical analysis was performed from three independent experiments each performed in triplicate. T-test was performed and comparisons were made between MAYV- versus CHIKV-infected cells. * represents *p* < 0.05, ** represents *p* < 0.005, **** represents *p* < 0.0001,

Because astrocytes are considered as the main cells responsible for general brain homeostasis and are often targeted following viral infection, for instance during arbovirus neuropathology [32], we decided to focus on this cell type to better characterize the potential mechanisms involved in MAYV-induced neuroinflammation. To better understand the effect of MAYV infection on astrocyte homeostasis, a total of 84 genes involved in antiviral response were screened in a RT-qPCR array following infection of primary human astrocytes by either MAYV or CHIKV (Figure 3). At 48 hpi, we observed differences in mRNA levels of several genes in MAYV- and CHIKV-infected astrocytes, as compared to mock-infected cells. This comparative analysis revealed that MAYV and CHIKV differentially induced the transcription of certain pattern recognition receptors (PRRs) in astrocytes. MAYV infection enhanced mRNA expression of *TLR3* and the RIG-I-like receptors (RLRs), *IFIH1* and *DDX58* (also known as MDA 5 and RIG-I, respectively), while CHIKV induced the transcription of *TLR7* and *DDX58* (Figure 3). The strong induction of type I IFN genes observed upon MAYV or CHIKV infection of astrocytes is in accordance with the recognition of both viruses by PRRs. Although increased IFN gene expression was observed after cell exposure by either MAYV or CHIKV, transcription of the IFN-stimulated gene (ISG) *ISG15* was strongly induced in MAYV-infected cells, compared to cells exposed to CHIKV, with a difference of approximately one log (Figure 3), most likely due to the strong increase in the *STAT1* gene in MAYV-infected cells. Moreover, a significant, albeit smaller, difference in the induction of *OAS2* and *MX1* transcripts between MAYV and CHIKV in infected astrocytes was observed as well. Caspase 10 (*CASP10*), a key element of the extrinsic pathway involved in apoptosis, was specifically upregulated by CHIKV and slightly downregulated by MAYV (Figure 3). The upregulation of cathepsin L1 (*CTSL1*) gene expression by MAYV contrasted with a three-fold stronger downregulation by CHIKV (Figure 3). Infection with either virus led to enhanced expression of the inflammatory chemokines *CCL5*, *CXCL10* and *CXCL11*, whereas mRNA expression levels of *IL-6*, *IL-12* and *IL-15* were only increased in MAYV-infected cells (Figure 3). Moreover, in agreement with the results from the RT^2^ Profiler PCR array analysis, MAYV induced the production of high levels of all the inflammatory mediators tested with the infected astrocytes, in contrast to CHIKV that induced the production of low levels of IL-12 and no IL-6 or IL-15, as compared to mock-infected cells (Figure 4). Taken together, these results show that at 48 hpi MAYV and CHIKV induce a different antiviral response in human astrocytes.

**Figure 5:**
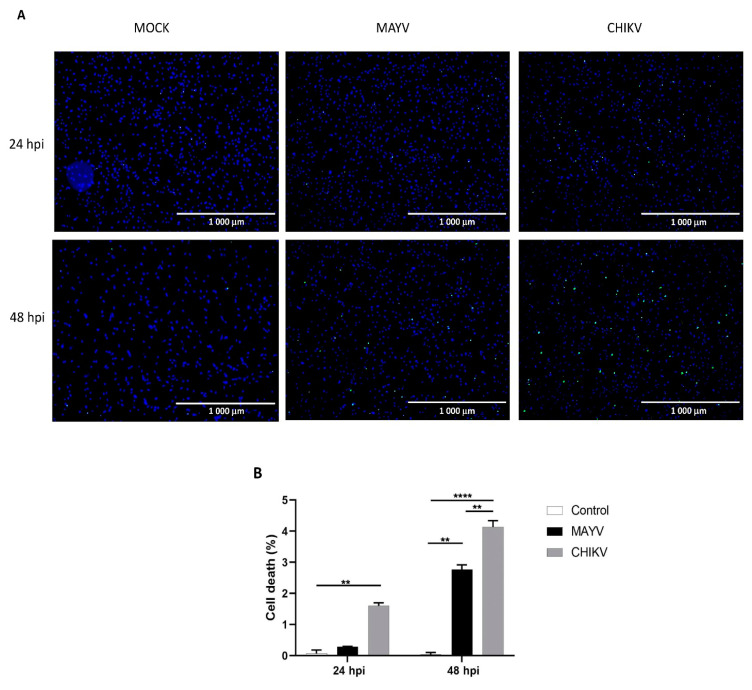
Cell death in primary human astrocytes following MAYV and CHIKV infection. (**A**) Live and dead cells were stained with NucBlue^®^ Live reagent (blue) and NucGreen^®^ Dead reagent (green) after infection with MAYV or CHIKV at MOI 5 at 24 and 48 hpi. Mock-infected cells were used as control. (**B**) Percentage of cell death. Mean values are represented. One-way ANOVA with Bonferroni’s post hoc test was performed. ** represents *p* < 0.005, **** represents *p* < 0.0001. The error bars represent the difference in the numbers of apoptotic cells counted in three fields. An average number of three fields were counted in the experiment (*n* = 2).

**Figure 6:**
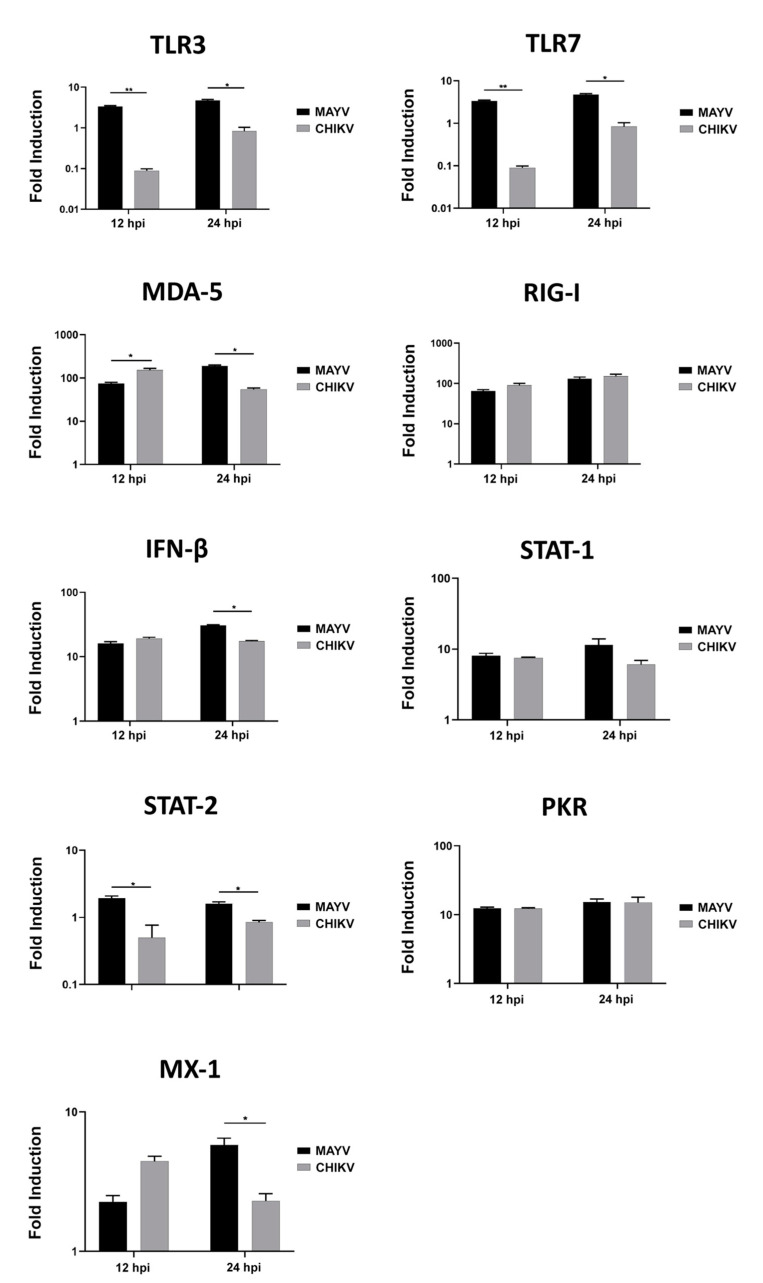
Modulation of antiviral gene expression by MAYV and CHIKV in human astrocytes. Astrocytes were cultured in the absence or presence of MAYV or CHIKV at an MOI of 5 and RNA was extracted 12 and 24 hpi, and innate immune gene expression was quantified by RT-PCR. Glyceraldehyde-3-phosphate dehydrogenase (GAPDH) was used for normalization. Modulation of antiviral response genes following MAYV or CHIKV infection is expressed as a function of that with mock-infected cells. The mean values are represented by each point, and two experiments were done, each performed in triplicate. Fold induction of transcript is shown. Wilcoxon–Mann–Whitney test was performed with a *p*-value significant when * *p*  <  0.05, ** *p*  <  0.01.

Since CHIKV has been reported to cause apoptosis in murine astrocytes, thereby significantly affecting the quantification of immune gene expression [25], we first tested whether MAYV or CHIKV were able to induce cell death in primary human astrocytes. Astrocytes were infected with MAYV or CHIKV, and cell viability was evaluated at 24 and 48 hpi. The results show that both MAYV and CHIKV induce negligible apoptosis in human astrocytes, with a maximum of 4% for CHIKV after 48 hpi (Figure 5), which contrasts with the abovementioned results in mouse astrocytes [25]. Therefore, it is very unlikely that the low percentage of virus-induced cell death induced by these arboviruses will affect the expression of innate immune genes in the present study. Moreover, to determine whether the induction of different innate immune genes by CHIKV occurs earlier than that induced by MAYV, we first checked the permissiveness of primary human astrocytes for MAYV or CHIKV at 12 and 24 hpi and, subsequently, quantified their expression levels at both two time points post-infection. Viral transcripts for both viruses increased in a time-dependent manner in infected cells, whereas MAYV appeared to infect human astrocytes less efficiently than CHIKV (Figure S1). The results also show that at these different post-infection times, there is a low induction of the genes selected for the analysis with the exception of the RIG-I-Like receptors, *MDA-5* and *RIG-I*, whose expression was up-regulated by both viruses (Figure 6). The expression of both *TLR3* and *TLR7* was found to remain more of less stable at early time points following viral infection. Furthermore, at 12 hpi CHIKV strongly induced *MDA-5* expression, to a greater extent than MAYV, with an inverse profile observed at 24 hpi. In contrast, the induction of *RIG-1* by MAYV and CHIKV remained unaltered irrespective of the virus strain and the time point post infection (Figure 6). Finally, a slight increase in the expression levels of *MX1* was observed at 24 hpi in MAYV-infected cells, as compared to CHIKV-infected cells (Figure 6).

### 3.3. Interferons Inhibit MAYV Replication in Primary Human Astrocytes

**Figure 7:**
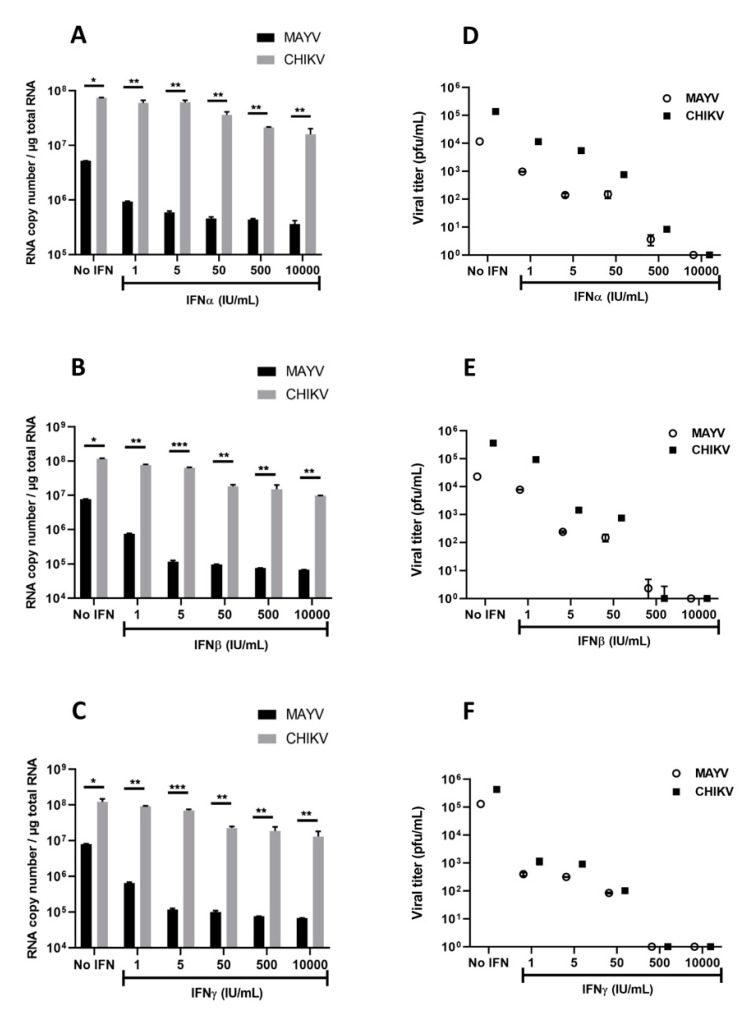
Interferons inhibit MAYV and CHIKV infection in primary human astrocytes. Cells, pre-treated in the absence or presence of various concentrations (1, 5, 50, 500, 10,000 IU/mL) of IFNs for 6 h, were infected with MAYV and CHIKV at MOI 1 for 48 h. Viral RNA was quantified by RT-qPCR (**A**–**C**), and infectious viral particles were measured using plaque assay (**D**–**F**). The experiments have been obtained from three independent experiments (*n* = 3) each performed in duplicate, and statistical analysis was carried out by using a one-way ANOVA test. Asterisks represent the statistically significant differences between the data (* *p* < 0.05, ** *p* < 0.005, *** *p* < 0.001) from three independent experiments.

Several studies on arbovirus have shown efficient viral replication inhibition by type I and type II IFNs [38,39]. Based on these studies, as well as the strong induction of *IFN-α* and *IFN-β* observed in MAYV- and CHIKV-infected human astrocytes, we aimed to evaluate their effects on MAYV and CHIKV replication in astrocytes. Cells were treated with different concentrations of type I or type II IFNs for 6h prior to infection with either MAYV or CHIKV (MOI 1) and viral replication (Figure 7A–C) and infectious viral particles (Figure 7D–F) were quantified. Each of these IFNs induced a dose-dependent inhibition of MAYV replication (Figure 7A–C), although pre-treatment of the cells with IFNs had less impact on CHIKV replication (Figure 7A–C). Indeed, only very high concentrations of IFNs led to a decrease in the amount of CHIKV RNA (Figure 7C). This reduction was approximately one log in cells pre-treated with IFN-β at concentrations greater than or equal to 50 IU/mL (Figure 7B). Inhibition of viral infection was also observed by plaque assay following pre-treatment with the type I and type II IFNs in MAYV-infected cells. We observed a strong and dose-dependent reduction in the release of infectious viral particles measured in supernatants of pre-treated MAYV-infected cells (Figure 7D–F). In concordance with the results of the viral RNA quantification, IFN pre-treatment resulted in a less pronounced decrease in the amount of infectious particles of CHIKV compared to cells infected under the same conditions with MAYV. These results demonstrate that IFNs are able to exert an antiviral effect on MAYV- and, to a lesser extent, CHIKV-infected astrocytes.

## 4. Discussion

Arthritogenic and neurotropic alphaviruses continue to spread around the world, leading to an increase in infections and, consequently, in the occurrence of associated arthralgia and neurological disorders [13,40]. Some arboviruses are now well described for their ability to access the CNS and cause short- and long-term neuropathology, potentially due to acute or chronic inflammatory states [40]. Neurological complications have been described in CHIKV-infected patients, in particular in children and the elderly. Moreover, no study has reported as yet on the neurotropism of MAYV, and the potential neuroinflammatory mechanisms resulting from infection with this alphavirus. The main focus of our study was to determine if CNS cells were permissive to MAYV and to compare the antiviral response elicited by MAYV and CHIKV in human astrocytes. 

In animal models, MAYV has been detected in the brain of young wild-type mice, not yet able to mount an efficient type I interferon response, as well as in type I IFN receptor-deficient mice [41]. Here, we show that the CHIKV infection rate is higher than that of MAYV in all CNS cell types tested. Our results also demonstrate for the first time and to the best of our knowledge that hNPCs are highly susceptible to both MAYV and CHIKV. This information is important because it has been shown that infection of hNPCs with Zika virus, another arbovirus, has been associated with the disruption of neurogenesis, resulting in serious neurological complications in neonates [42,43]. Further in-depth studies are needed in newborns in areas with an endemic or epidemic circulation of both viruses.

Pericytes play a crucial role in the neurovascular unit (NVU), including the regulation of BBB permeability and neuroinflammation [44], although it is as yet unclear whether infection of the latter cells by CHIKV could contribute to the neuroinvasion of this virus and could have potential neurological consequences following MAYV infection as well. Astrocytes support general brain homeostasis through the regulation of various mechanisms including synaptic transmission, BBB function or neuroinflammation [30]. Numerous neurotropic arbovirus from the Flavivirus family reportedly target astrocytes during infection and elicit a potent neuroinflammatory environment that is thought to lead, at least in part, to neurological impairment. CHIKV infection has been shown to lead to innate immune activation of astrocytes in cynomolgus macaques [26] and the susceptibility of mouse astrocytes as well as U-87 MG human astrocytic cell line to CHIKV in vitro has been described [25,45]. Infection of the brain of macaques with CHIKV also resulted in morphologic changes in astrocytes, suggesting that these modifications could affect the interaction between astrocytes and the neighboring cells present in the NVU such as microvascular endothelial cells and neurons. In the present study, we have analyzed the ability of primary human astrocytes to induce an innate antiviral immune response as a result of MAYV infection. Overall, the modulation of the immune gene expression profile in MAYV-infected astrocytes, analyzed at 48 hpi, was more extensive, in comparison to that in cells infected by CHIKV. The innate immune response in immune-competent cells, including those in the CNS, is activated by the upregulation of a high diversity of genes [26,46]. PRRs such as TLRs are present in brain cells [46]. Surprisingly, the expression of *TLR3,* and not *TLR7*, was found to be upregulated by MAYV, albeit inversely by CHIKV. This observation highlights the difference in the signaling pathways that are triggered by these viruses following their recognition by TLRs expressed in astrocytes. The observed difference could be explained by the time post-infection at which the analysis was carried out, suggesting that CHIKV activates TLR3 more rapidly than MAYV. However, our results on the quantification of *TLR3* transcripts at 12 and 24 hpi fail to show a strong induction of this gene by CHIKV in primary human astrocytes, as compared to its level in mock-infected conditions. In the study by Priya et al. that was carried out in one-week-old mice infected with CHIKV, the virus induced an upregulation of *TLR3* expression in the brain, although the authors did not specify the nature of the infected cells [46]. The importance of the TLR3 signaling pathway in CHIKV infection has previously been demonstrated by the observation that viral infection resulted in an increase in viral load by about 100 times in deficient TLR3 mice [47]. Similarly, some genetic variants of TLR7 have been shown to enhance susceptibility to CHIKV infection among individuals suggesting that these variants could be used as biomarkers amongst healthy individuals to predict susceptibility to CHIKV infection [48].

It has previously been shown that CHIKV infection does not modulate the expression of TLR2 levels in astrocytes, but an upregulation was observed in areas that were GFAP immunoregulative following challenge with CHIKV in cynomolgus macaques at 35 dpi, suggesting that cells, other than astrocytes, might express this TLR [26]. Our finding corroborates this result to the extent that we did not observe any modulation of *TLR2* in our analyses. In our experimental conditions, we also observed a strong induction of the PRRs *IFIH1* and *DDX58* transcripts by MAYV, as compared to that by CHIKV. However, unlike *TLR3* and *TLR7*, these two viruses significantly increase the level of expression of *IFIH1* and *DDX58*. Cells have developed several mechanisms to limit viral propagation, among others, the overexpression of chemokines allowing recruitment of immune cells. However, in the CNS, uncontrolled chemokine production and immune cell recruitment can be detrimental for brain functions and lead to neurological disorders [35]. Here, we show that CXCL10, CXCL11 and CCL5 known to be expressed in the CNS during various viral infections including those by encephalitic arboviruses [49,50] were upregulated by either virus, pointing to the induction of a strong inflammatory response. It is important to note, however, that chemokines, such as CXCL10, have multiple effects in virus-induced neuropathy, since on the one hand, it has been reported to be neuroprotective, whereas on the other hand, it exerts neuro-pathogenic potential by triggering apoptosis [51,52,53].

IFNs are cytokines known to interfere with the viral cycle precisely at the replication stage and consequently promote an antiviral state. The main IFNs produced following a viral infection are type I and II IFNs that signal through the JAK-STAT pathway resulting in the induction of several ISGs. Results from an experimental mouse model showed that several ISGs, among which is ISG15, were upregulated following infection of the brain by CHIKV [46]. Our results are consistent with the latter study showing an increase in *ISG15* expression in CHIKV-infected astrocytes, although this level of expression was lower than that observed in MAYV-infected cells. Surprisingly, the expression of *IRF3*, known to play an important role in the induction of IFNβ, was not modulated by CHIKV, but, instead, was enhanced by MAYV infection. The induction of *IFNβ* and not *IRF3* by CHIKV in the present study corroborates a previous study on SINV [54], highlighting the fact that signaling via the IRF3 pathway is not strictly required for IFNβ induction in the CNS. Mice lacking *IRF3* have been shown to survive from SINV infection with persistent affection of the CNS. It has previously been shown that IL-6 production was enhanced following brain infection by CHIKV [46]. IL-6 is produced by various brain cells, including astrocytes, and is likely to be important for BBB integrity [55,56,57]. This inflammatory mediator has also previously been detected in CHIKV-induced persistent arthralgia [58], and we have recently shown that infection by MAYV of primary human chondrocytes leads to IL-6 production [36].

Some studies have shown that arboviruses, including alphaviruses, use several strategies to escape from the action of IFNs by a shut-off of the host cell macromolecular synthesis or activation [59,60]. Moreover, it has been demonstrated that the deficiency in type I IFN receptors in the CNS enhances the neuropathogenesis induced by several viruses [61,62]. The ability of astrocytes to upregulate type 1 IFNs upon flavivirus infection has been previously demonstrated [61]. In the present study, we show that pre-treatment of astrocytes with IFNs prior to infection could restrict MAYV replication and, to a lesser extent, CHIKV. This result corroborates a previous study highlighting the fact that CHIKV was rather insensitive to IFN pre-treatment that had very little effect on the production of infectious viral particles on Vero cells [59]. The same observation was also reported in a study conducted on primary mouse cortical neurons with other alphaviruses such as Venezuelan equine encephalitis and SINV [63], suggesting a difference in IFN sensitivity depending on the nature of the virus. Differences in the immune response profile between MAYV- and CHIKV-infected cells have also been demonstrated in monocytes [64]. MAYV was shown to mostly activate an anti-inflammatory response, whereas CHIKV activates a Th1 and Th17 response [64]. The neurovirulence of CHIKV can be partially explained by this resistance to IFN and the action of its nonstructural protein 2 (nsp2) and envelope proteins, which strongly inhibit the activation of the IFNβ promotor [65]. CHIKV nsp2 has also been shown to impact type I IFN response by promoting the nuclear export of STAT1 necessary for the activation of the transcription of several antiviral ISGs [66]. These factors could contribute to the severity of infection due to CHIKV in patients with arthritis and the ability of the virus to spread into the brain. In contrast, the amount of IFN produced by MAYV-infected cells and the susceptibility of the virus to the IFN response may be sufficient to restrict MAYV infection. This could explain why, despite the increasing number of reported outbreaks, very few patients with neurological complications due to MAYV have been reported. 

In the present study, we show that MAYV can efficiently replicate in CNS cells, albeit at a lower rate than CHIKV, which could be explained by a stronger antiviral response because of the observed higher modulation of antiviral genes in MAYV-infected astrocytes and potent inhibition of viral replication following type I IFN pre-treatment. The difference in infectivity between MAYV and CHIKV could also be related in part to the recognition by these viruses of specific attachment/host factors. Indeed, it has recently been shown that the four-and-a-half LIM domain protein 1 (FHL1) is a host factor required for the infection of fibroblasts and skeletal muscles cells by CHIKV but that the absence of the latter did not prevent the entry of MAYV into these cells [67]. It would also be interesting to know whether brain microvascular endothelial cells are permissive to MAYV as well, given that these cells are part of the BBB and important for the first line of defense against invasion of the CNS by pathogens [68]. More studies need to be carried out to identify different access of MAYV to gain the CNS and to determine whether differences in neurotropism or neuropathology exist between different MAYV genotypes. Indeed, this possibility is supported by the results obtained in our laboratory showing significant differences in the production of viral RNA in primary human astrocytes depending on the MAYV genotype used for infection (unpublished data). The implementation of several study models and techniques will permit us to determine the multiple mechanisms that are involved in the potential neurotropism of this emerging arbovirus. 

## Data Availability

Not applicable.

## Supplementary Materials

The following are available online at https://www.mdpi.com/1999-4915/13/3/465/s1, Figure S1: Primary human astrocytes are permissive to MAYV and CHIKV at early time points post-infection. Table S1: List of primers used in the study.
