# Supplementary material for: Mayaro Virus Infects Human Brain Cells and Induces a Potent Antiviral Response in Human Astrocytes
Source: Viruses. 2021 Mar 11;13(3):465. doi: 10.3390/v13030465 (PMC8001792; doi:10.3390/v13030465)
Supplement: Supplementary file 1 [file viruses-13-00465-s001.pdf]

viruses

MDPI

Supplementary Materials: Mayaro Virus Infects Human Brain Cells and Induces a Potent Antiviral Response in Human Astrocytes

Table S1. List of primers used in the study.

Name	Sequence (5'–3')
TLR3	TGGGACCAAGGCAAAGGAGT
	TTCTCTTGGTTGGGCCACCT
TLR7	AATGTCACAGCCGTCCCTAC
	TTATTTTTACACGGCGCACA
MDA-5	GCCATTGCAGATGCAACCAG
	TTGCGATTTCCTTCTTTTGCAG
RIG-I	AGTGAGCATGCACGAATGAA
	GGGATCCCTGGAAACACTTT
IFN-◎	GACGCCGCATTGACCATCTA
	TTGGCCTTCAGGTAATGCAGAA
STAT1	AAAAATTTTCCCTTCTGGCTTTGCATTGAA
	GCAGCCATGACTTTGTAATTGCGAATGAT
STAT2	GAAAACTTTGACCCCCGAGAA
	CGTGAAGCTGATGATGTGCAG
PKR	TGGAAAGCGAACAAGGAGTAAG
	CCATCCCGTAGGTCTGTG
MX1	GCGGGCTGTGGATATGCT
	TTTATCGAAACATCTGTGAAAGCAA
GAPDH	TCGGAGTCAACGGATTTGGT
	TCGCCCCACTTGATTTTGGA

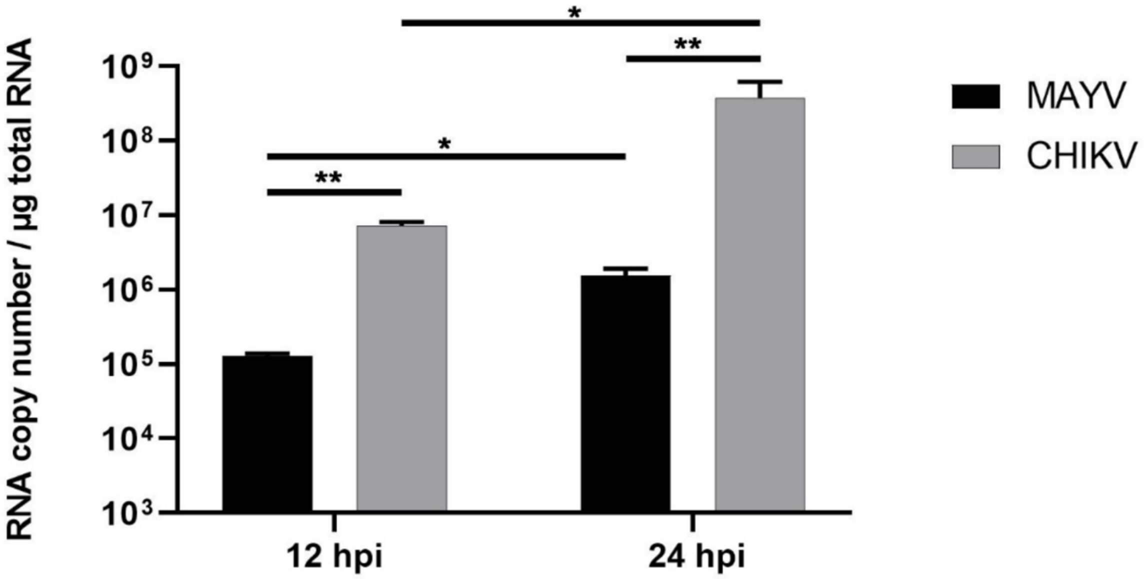

Figure S1. Primary human astrocytes are permissive to MAYV and CHIKV at early time points post-infection. Cells were infected with either MAYV or CHIKV at MOI 5 at 12 and 24 hpi. RT-qPCR were performed to measure viral RNA level in infected cells. Statistical analyses were done from two independent experiments each performed in triplicate. Comparisons between the data at the indicated time points using one-way ANOVA test. * represents $p < 0.05$, ** represents $p < 0.01$.
